# Introduction to the Special Issue on Cyberbullying and Cybervictimization

**DOI:** 10.3390/ejihpe12060045

**Published:** 2022-06-03

**Authors:** Cecilia Ruiz-Esteban, Inmaculada Méndez, Aitana Fernández-Sogorb

**Affiliations:** 1 Department of Evolutionary Developmental and Educational Psychology, Faculty of Psychology, University of Murcia, 30100 Murcia, Spain; cruiz@um.es (C.R.-E.); inmamendez@um.es (I.M.); 2 Department of General Didactic and Specific Didactics, Faculty of Education, University of Alicante, 03690 Alicante, Spain

Nowadays, the Internet is commonly used by students for both social activities and the academic field. In this context, problematic mobile phone or Internet use is among the most common risks that have proven to be associated with bullying and cyberbullying [1,2]. The persistent behavior of trying to injure a helpless victim, which can be carried out by means of new technologies, has serious physical, psychological, and educational consequences [3]. This problem affects not only students who participate in situations of school violence, but also teachers, families, and society. For this reason, it is necessary to deepen the knowledge on cyberbullying and cybervictimization by examining students’ characteristics and listening to stakeholder groups’ perceptions.

This Special Issue presents a compilation of six studies that try to identify protective, contextual, and risk factors of school violence and, specifically, of cyberbullying and cybervictimization in European and North American contexts: three out of the six works were conducted in Spain and the remaining three studies in Italy, United States, and Canada. In addition to the cultural diversity examined, this Special Issue manages to understand cyberbullying throughout three evolutionary stages: childhood, adolescence, and adulthood. 

Regarding childhood, two works were carried out with Spanish and Italian samples. On the one hand, the Italian study was conducted with Sicilian primary school students to examine the predictive capacity of sociodemographic variables (gender and age) and mechanisms of moral disengagement (moral justification, euphemistic language, advantageous comparison, displacement of responsibility, disclosure of responsibility, distorted consequences, blame attribution, and dehumanization) on the manifestation of three different roles that are characteristic of school violence: victims, aggressors, and bystanders [4]. On the other hand, the Spanish work was carried out with Spanish primary school students to analyze whether socio-emotional competencies (relationship skills, self-management, and social awareness) can affect the overlap of off- and online bullying in those who manifest the role of victims and to examine their gender differences [5]. The results of these works reveal that European children who suffer victimization tend to use moral justification and show low self-management, in the case of girls, and poor relationship skills, in the case of boys. Furthermore, dehumanization is a predictor of the role of both the aggressor and the bystander. These findings offer rigorous information on the characteristics of victims, aggressors, and bystanders, which can be useful for readers who are interested in promoting adaptive social relationships in centers of primary education.

As for adolescence, research was conducted with samples from Spain and the United States. The former study focused specifically on intrapersonal emotional intelligence [6] and the latter focused on internalizing symptoms [7]. Similar results were obtained since Spanish adolescents with poor emotional regulation tended to manifest problems related to depression, anxiety, or violence and disruptive behaviors, and American adolescents who had the role of bystanders manifested more problems related to depression, anxiety, and somatic symptoms than those who did not have that role. Therefore, adolescents from different cultures share a risk profile regarding the manifestation of psychopathological symptoms and the engagement in bullying and cyberbullying roles. This empirical evidence encourages readers to consider the mental health of secondary education students who participate in situations of school violence.

With respect to adulthood, this Special Issue includes two works centered on solutions to cyberbullying suggested by college students from Canada [8] and characteristic features of criminal mediation in Spain [9]. The former presents as a relevant solution the development of a sense of belonging among college students, which should be achieved with the collaboration of all university stakeholders. The latter study shows that the adoption of agreements tends to be characterized by moral content regardless of the age of the respondent and the complainant. However, it was found that young adults constitute the group with more problems of physical violence. These results may lead the reader to reflect on the importance of mediation and students’ integration inside and outside educational system.

Several practical implications are offered by this Special Issue to improve mental health and interpersonal relationships throughout the three evolutionary stages examined. During childhood and adolescence, authors suggest applying programs centered on the development of empathy and inter- and intrapersonal emotional intelligence to prevent both bullying and cyberbullying or to reduce their impact on students’ wellbeing [4,5]. According to Garaigordobil [6] and Doumas and Midgett [7], psychopathological symptoms are specially worrying in adolescents, so it is recommended that interventions targeting learners of secondary education include strategies to manage, for example, depression and anxiety. Regarding adulthood, the study by Faucher, Cassidy, and Jackson [8] encourages researchers to listen to stakeholder groups at university, such as students or staff. Thus, similarities in their perceptions about possible solutions provide accurate information to design effective programs to address cyberbullying in higher education settings. Finally, Matás, Méndez, Esteban, and Soto [9] suggest implementing relationship care education at all educational stages not only to prevent violence, but also to facilitate mediation in adulthood.

In sum, this monograph allows us to conclude that cyberbullying and cybervictimization are a worldwide problem that needs to be address in primary, secondary, and higher education settings. Furthermore, students’ characteristics, according to gender and the three different roles that intervene in school violence situations (i.e., victims, aggressors, and bystanders), must be also considered. As a consequence, these findings can form the basis for collaboration among researchers and professionals of the Health, Psychology, and Education fields in the design of cyberbullying prevention and intervention programs.
